# Multilevel competing risks in the evaluation of nosocomial infections: time to move on from proportional hazards and even from hazards altogether

**DOI:** 10.1186/cc13892

**Published:** 2014-05-27

**Authors:** Alvaro Muñoz, Nicole Mongilardi, William Checkley

**Affiliations:** 1 Department of Epidemiology, Bloomberg School of Public Health, Johns Hopkins University, 615 N Wolfe St, Suite E7648, Baltimore, MD 21205, USA; 2 Division of Pulmonary and Critical Care, School of Medicine, Johns Hopkins University, 1800 Orleans Avenue, Suite 9121, Baltimore, MD 21205, USA

## Abstract

A competing risk is an event (for example, death in the ICU) that hinders the occurrence of an event of interest (for example, nosocomial infection in the ICU) and it is a common issue in many critical care studies. Not accounting for a competing event may affect how results related to a primary event of interest are interpreted. In the previous issue of *Critical Care*, Wolkewitz and colleagues extended traditional models for competing risks to include random effects as a means to quantify heterogeneity among ICUs. Reported results from their analyses based on cause-specific hazards and on sub-hazards of the cumulative incidence function were indicative of lack of proportionality of these hazards over time. Here, we argue that proportionality of hazards can be problematic in competing-risk problems and analyses must consider time by covariate interactions as a default. Moreover, since hazards in competing risks make it difficult to disentangle the effects of frequency and timing of the competing events, their interpretation can be murky. Use of mixtures of flexible and succinct parametric time-to-event models for competing risks permits disentanglement of the frequency and timing at the price of requiring stronger data and a higher number of parameters. We used data from a clinical trial on fluid management strategies for patients with acute respiratory distress syndrome to support our recommendations.

## 

In the previous issue of *Critical Care*, Wolkewitz and colleagues [1] analyzed data from a multi-center cohort study of 159 ICUs and 109,202 admissions to better understand how both patient-level and ICU-level characteristics affect nosocomial infections (NIs). NIs are a leading cause of death in the ICU despite being largely preventable [2]. Reductions of up to 70% have been achieved via infection control strategies for specific NIs [3], but this has not been universal across settings. Thus, it is important to define both high-risk patients and high-risk settings and identify interventions that prevent NIs, for which data analysis methods to determine risk factors for specific events are pertinent. Wolkewitz and colleagues [1] acknowledge the difficulty of the problem caused by heterogeneity across ICUs and the importance of competing risks when analyzing risk factors for NIs and present a statistical approach to handle both of these complexities.

## Heterogeneity across intensive care units

In recent years, increasing attention has been given to the role of ICU organization and processes of care on patient-centered outcomes [4-9]. Some of these previous investigations have also recognized substantial heterogeneity in ICU organization [4,9] that can contribute to variations in rates of mortality of critically ill patients across hospitals and may help explain why some interventions achieve improvements only in specific settings. By using a multilevel approach to analyze patient-level and ICU-level characteristics, Wolkewitz and colleagues thereby avoid loss of valuable information that may help explain differences in clinical outcomes.

## Competing risks in critical care

Competing risks are important in the ICU because a large proportion of patients may be discharged or die before developing the event of interest (for example, NIs). To account for competing risks, Wolkewitz and colleagues [1] extended methods to compare cause-specific hazards (CSHs) and sub-hazards (SUBHs) of the cumulative incidence functions between study groups [10] by incorporating random effects to quantify heterogeneity. However, despite wide recognition that proportionality of hazards does not occur in most situations, their results are presented under such an assumption.

## Time to move on from proportionality of hazards

Proportionality of hazards has additional hurdles in competing risks [11]. In the case of two competing risks, proportionality cannot hold simultaneously for all CSHs and SUBHs [12]. Specifically, at most two of the four (two events and two types of hazards) can be proportional but without restriction of which two [11]. Even in the case in which two of the four hazards are proportional, there is strong tethering between the hazard ratios [11]. In particular, when the CSHs and SUBHs for a given event type simultaneously fulfill proportionality, the two hazard ratios must be equal and this fact holds regardless of the number of competing risks. A consequence of this result is that analyses reporting CSH and SUBH ratios with different values divulge a lack of proportionality in at least one of the hazard types, as is the case in the analysis by Wolkewitz and colleagues [1] for number of beds, Acute Physiology and Chronic Health Evaluation II score, days in hospital, type of diagnosis and trauma as presented in their Table 2. Fine and Gray [13] anticipated that a lack of proportionality would be common in competing-risk problems. While it is possible to test for a lack of proportionality, power is often limited in most situations (for example, short follow-up time or heavy censoring). Thus, default analyses should include covariate and time interactions which can be easily calculated and depicted [14].

## Even time to move on from hazards altogether

Hazards as a metric of disease occurrence along with semi-parametric methods have provided a means to understanding many diseases. However, hazards have limitations [15] that become compounded in competing-risk problems: CSHs and SUBHs are decoupled; CSHs lack specificity as they are strongly influenced by the competing event; SUBHs are specific but they are intrinsically tethered because their cumulative incidences must add up to one; both CSHs and SUBHs combine frequency and timing of events, making it difficult to identify exposures that modify only the timing of an event but not the frequency.

**Figure 1:**
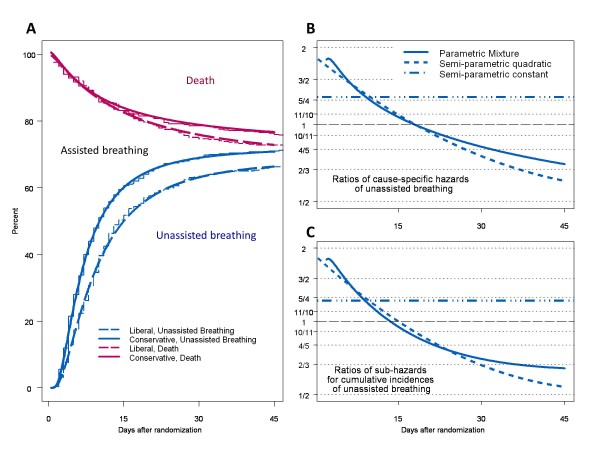
**Parametric and non-parametric estimates of the cumulative percentages of ventilated patients who either achieved unassisted breathing or died after randomization in the fluid management trial.** Estimates are stratified by study group **(A)** and ratios of conservative to liberal strategies of cause-specific hazards **(B)** and sub-hazards **(C)** of unassisted breathing. In all of these models, death before unassisted breathing is the competing risk. For panel (A) (Adapted with permission from Lippincott Williams and Wilkins/Wolters Kluwer Health: *Epidemiology*[17], copyright 2010), parametric estimates are represented with continuous lines and non-parametric estimates are represented with steps. The continuous lines give evidence to the goodness-of-fit of mixtures of generalized gamma distributions for the timing of the two events in both treatment groups.

Approaching the analysis of competing risks as a mixture of flexible and succinct parametric distributions overcomes limitations of hazards and can provide enlightening insights [16,17]. To illustrate this, in Figure 1A we show the cumulative incidences among individuals receiving mechanical ventilation of achieving unassisted breathing and of dying in the hospital at different days after being randomly assigned to receive either a liberal or conservative fluid management strategy [17]. The core inference from this analysis was the beneficial effect of conservative fluid management by significantly reducing the days to achieve unassisted breathing but having no effect on the overall frequency of unassisted breathing and on timing of death [17]. This is a very specific finding. In contrast, Figures 1B and 1C present the inappropriate and oversimplistic summary provided by the analyses under proportional-hazards assumptions (horizontal lines with dashes and dots) and the agreement of a strong downward trend of the hazard ratios from the mixture of parametric distributions (solid curves) and the semi-parametric analysis permitting treatment and time interactions (short-dashed curves). The inference from both analyses and from the two hazard types would be that conservative fluid management increases the hazards of unassisted breathing in the first 2 weeks but precludes unassisted breathing among those who remained mechanically ventilated for longer. Although these inferences are not incorrect, they are murky. Mixture models enable the disentanglement of frequency and timing in competing risks. However, they require strong data and may demand large numbers of parameters. The data in Wolkewitz and colleagues are ‘as strong as they get’, and it remains of interest to explore new approaches beyond the novel inclusion of random effects that the authors have put forward.

## Abbreviations

CSH: Cause-specific hazard; NI: Nosocomial infection; SUBH: Sub-hazard.

## Competing interests

The authors declare that they have no competing interests.

## Authors’ contributions

AM, NM, and WC reviewed and approved the final version of the commentary. All authors read and approved the final manuscript.
